# Keeping Tabs on HABs: New Tools for Detecting, Monitoring, and Preventing Harmful Algal Blooms

**DOI:** 10.1289/ehp.122-A206

**Published:** 2014-08-01

**Authors:** Nate Seltenrich

**Affiliations:** Nate Seltenrich covers science and the environment from Petaluma, CA. His work has appeared in *High Country News*, *Sierra*, *Yale Environment 360*, *Earth Island Journal*, and other regional and national publications.

**Figure:**
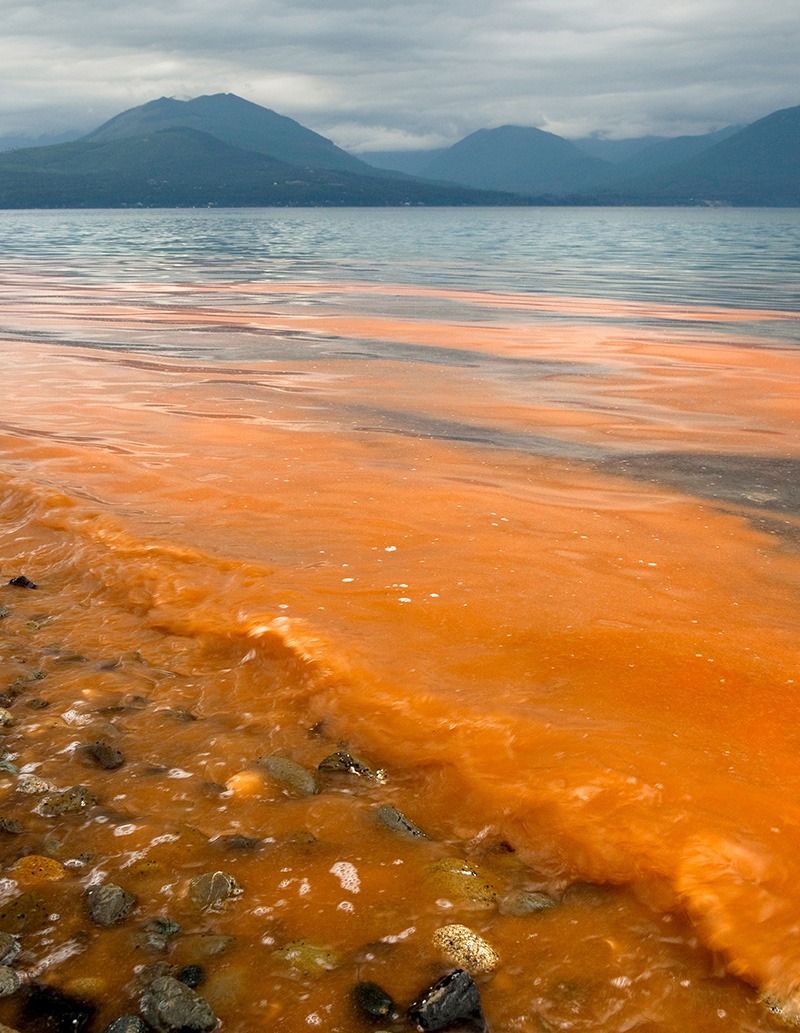
A red tide rolls ashore in Puget Sound, Washington. Environmental changes, heightened awareness, and improved detection have increased the number of HABs reported each year. © Purestock/Alamy

Thomas Hoover once worked as a nuclear reactor operator for the U.S. Navy and has installed radar-absorbing technologies on its warships. He has helped design communications satellites and repaired explosive ordnance disposal robots in Iraq. Now his expertise is aimed at another adversary: harmful algal blooms (HABs).

Hoover and his colleagues at the Monterey Bay Aquarium Research Institute (MBARI) are building what may be the world’s most powerful and adaptable HAB detection device yet, offering a new range of capabilities to researchers.^1^ This novel approach to detecting HABs and mapping their size and toxicity is the product of a team of engineers and scientists from MBARI and their collaborators at the University of Washington at Seattle, Arizona State University, the University of Maine at Orono, and the National Oceanic and Atmospheric Administration (NOAA) Marine Biotoxins Program in Charleston, South Carolina.

Yet it’s only one example among many emerging technologies that are transforming the field by allowing scientists to study HAB ecology in both fresh- and saltwater environments more precisely and efficiently than ever before. Improved communications capabilities, higher-resolution satellite imagery, and smaller, more powerful sensors have all contributed to significant advances in recent years, just as HAB activity is escalating worldwide.^2^

HABs occur naturally, but in recent years nutrient-rich agricultural runoff, transport of HAB species via ship ballast water, coastal aquaculture farms (which both introduce nutrients and are in turn threatened by blooms), and climate change appear to have contributed to an expansion and intensification of HAB activity worldwide. Heightened awareness and improved detection of HABs also have led to increased numbers of reported events in recent years.^3^^,^^4^^,^^5^

HABs come in a variety of forms and cause harm in different ways. Concentrations of nontoxic algae can deplete waters of oxygen and irritate fish gills, while toxin-producing species can accumulate quickly and seemingly without warning in fresh- and saltwater bodies, posing a threat to humans, fish (including some commercial species), mammals, and birds.^2^

Humans are exposed to algal toxins in three primary ways: when we drink contaminated water, when we eat fish or shellfish in which toxins have accumulated, and when we inhale aerosolized toxins.^6^^,^^7^ Health effects can include gastrointestinal, neurological, dermal, and respiratory symptoms, depending on the route of exposure and the type of toxin. In some cases, exposure can be fatal.^8^

Accurately tracking and forecasting HABs, as NOAA and others already do in some areas, can dramatically reduce their impacts on human health, fisheries, and economies. But growing pressure to cut costs means new ways must be found to achieve these goals. Investigators also want to improve forecasts by utilizing new instruments that can autonomously collect high-frequency HAB and environmental data, minimizing the need for expensive survey vessels and human sampling and analysis.^9^^,^^10^ Being able to see blooms coming is “the Holy Grail for all HAB research,” says Raphael Kudela, a phytoplankton ecologist at the University of California, Santa Cruz.

## Studying HAB Ecology

Concurrent with the development of their new state-of-the-art HAB detection device, MBARI researchers are hand-building a long-range autonomous underwater vehicle named *Makai*. A 28-inch section of the 10-by-1-foot missile-shaped robot is reserved for the device, the third generation of what’s known as an Environmental Sample Processor (ESP). The final assembly will contain about twice as many components as a car and will be able to travel thousands of kilometers at sea, regularly communicating with shore as it goes, while performing a battery of scientific tests, says Hoover.

MBARI president and chief executive officer Chris Scholin is recognized among oceanographers for inventing and developing the ESP. The first iteration of the device debuted in the Gulf of Maine in 2001, where it was deployed to detect *Alexandrium fundyense*,^11^ an algal species whose toxins cause an illness known as paralytic shellfish poisoning.^12^

**Figure:**
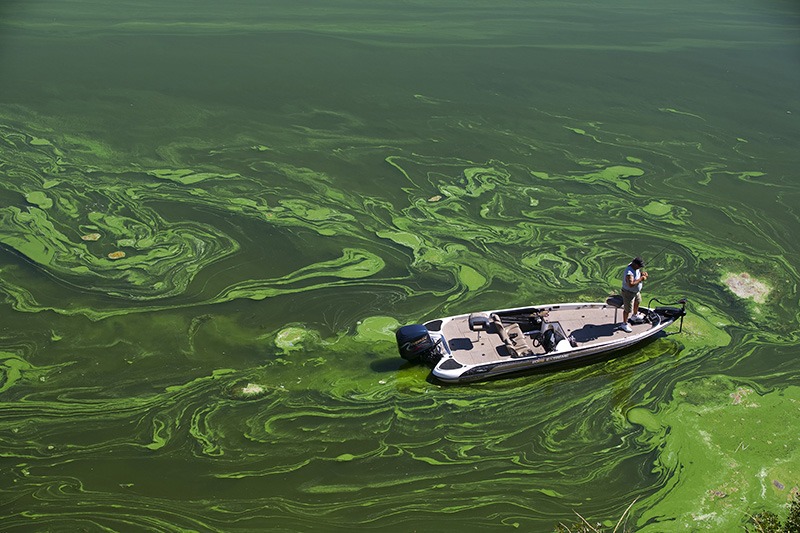
A cyanobacterial bloom on the Klamath River near Copco, California, swirls around the boat of a sport fisherman, who told the photographer, “Anyone who sees this [photo] is gonna say, ‘That guy’s stupid,’ but they ought to come up and try it. The algae provides a cover for the fish.” However good or bad cyanobacterial blooms may be for fishing, they also produce toxins that can irritate eyes, skin, lungs, and the gastrointestinal tract, and may even cause nerve damage or death at high exposures.^7^ © David McLain/Aurora Photos/Corbis

The current (second-generation) ESP is often referred to as a “laboratory in a can”; it’s roughly the size of a 50-gallon drum. It arrived in 2006 and has seen sustained HAB-related deployments in a range of settings, including New Zealand for an aquaculture management and water-quality research project;^13^ the U.S. Northeast, where protecting the public from paralytic shellfish poisoning remains an urgent concern; and the Pacific Northwest, where subsistence and recreational razor clam harvests are threatened by the alga *Pseudo-nitzschia* and its toxin, domoic acid, which causes amnesic shellfish poisoning.^14^ Sixteen second-generation ESPs exist in the world today, with six built by MBARI and the rest by Massachusetts-based McLane Research Laboratories, Inc.

Like the newest device, the second-generation ESP is capable of autonomously sampling seawater, measuring water quality, filtering particles from the water, using DNA analysis to detect microorganisms contained in those particles, assessing the presence and concentration of algal toxins, and testing or storing the particle-laden filters for subsequent analysis, all while receiving remote instruction from its operators and returning data on what it finds. Its size, however, limits its transportability and deployment options primarily to moorings, pier mountings, and surface floats. It cannot follow HABs as they move with waves and ocean currents or explore them in three dimensions.

That is why the third-generation ESP, which will be ready for testing later in 2014 and commercially available within three to four years, represents a leap forward in HAB monitoring. Offering similar capabilities to its predecessor in a more user-friendly package about one-tenth of the size—and, ideally, for a lower price, Scholin says—the new device will be able to detect and physically track a developing bloom as it moves through the ocean. “With respect to harmful algae, we think of it as being akin to ‘sending the dogs out,’” says Scholin.

The data to be collected by the third-generation ESP should not only provide researchers more information about HAB ecology in order to better forecast blooms on a seasonal scale, but also help them identify and track specific events before they come close to shore. That means beaches and fisheries can be closed before it’s too late, but for no longer than necessary.

The new ESP can be fitted into backpacks or small mobile labs for field use in a variety of environments, Scholin says. He believes freshwater applications will be the next frontier for both second- and third-generation ESPs. This will require novel tests and sensors for freshwater algae and their toxins, which he says independent teams around the world are currently developing. The devices could provide new levels of detection capabilities to protect drinking-water sources and recreational users of lakes and reservoirs.

## Getting a Handle on HABs

When most people think of HABs, they think of “red tides,” although in reality algal blooms can come in a rainbow of colors, and not all blooms are toxic. Coastal waters and freshwater lakes nationwide are subject to nine distinct types of HAB phenomena, with most endemic to certain geographic areas.^15^ All 50 states are affected, including virtually the entire U.S. coastline and freshwater bodies from the Great Lakes down to small ponds.^16^ And HABs can be surprisingly expensive. They have been estimated—conservatively, NOAA stresses^17^—to cost coastal communities in the United States an average of $82 million per year.^18^ Human health impacts due to lost wages and costs of medical treatment and investigation accounted for $37 million of that estimate.

**Figure:**
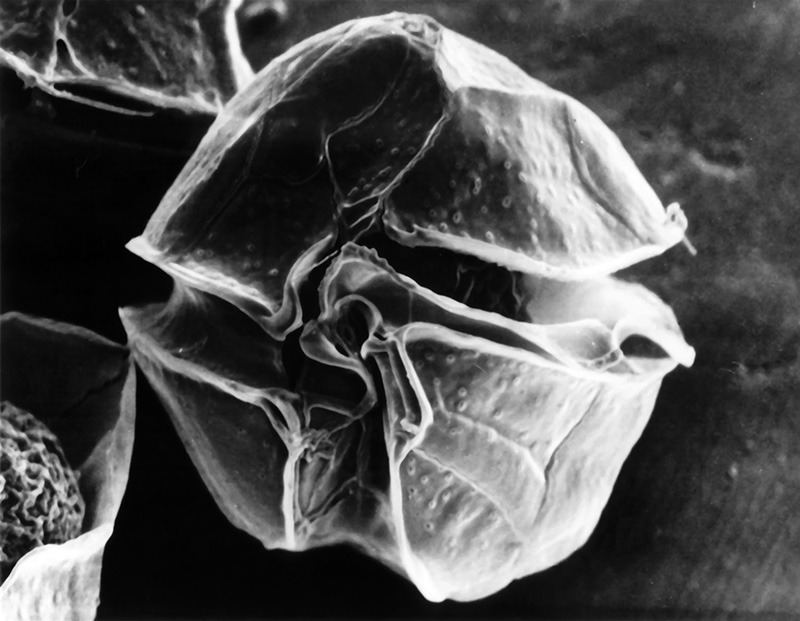
Toxins produced by *Alexandrium fundyense* accumulate in filter-feeding shellfish including clams, oysters, and mussels. These toxins can cause an illness called paralytic shellfish poisoning in people who eat contaminated shellfish. © A. L. Loeblich III/WHO

**Figure:**
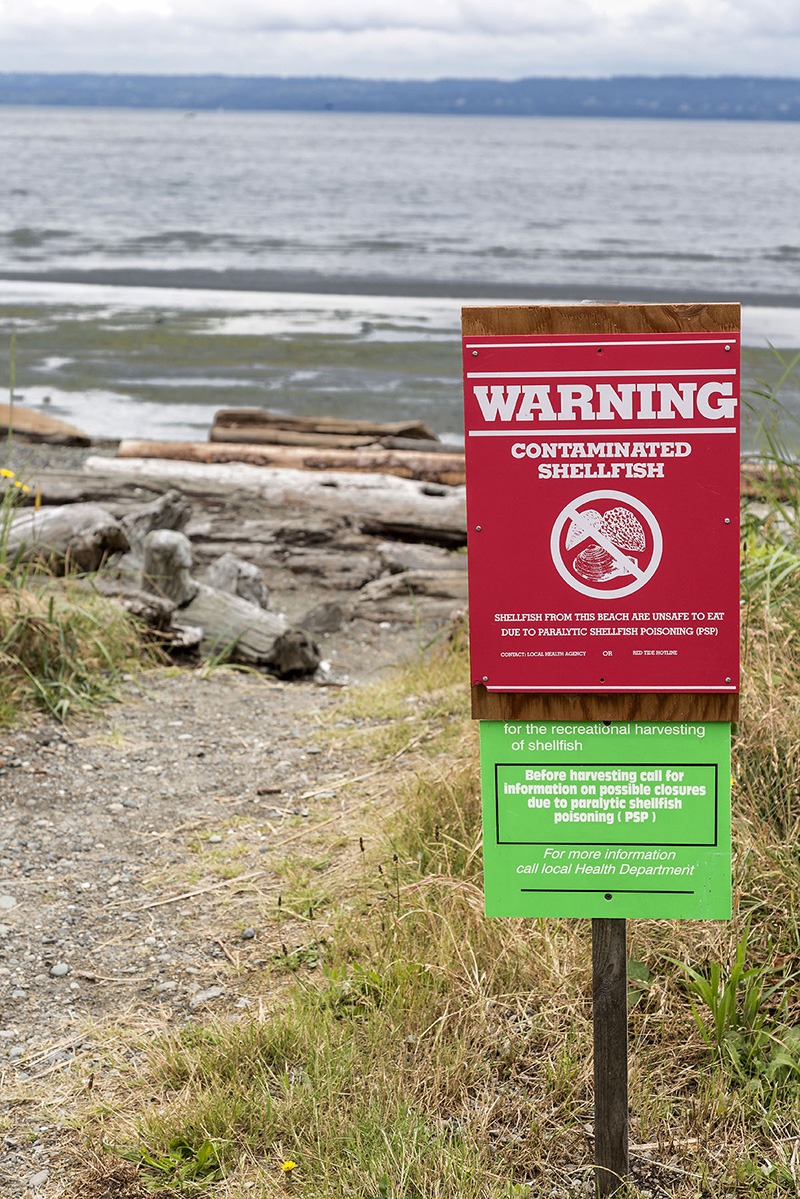
New HAB detection measures aim to reduce the amount of time that shellfish beds are closed unnecessarily. © Steve Froebe/iStock

Assessing the true extent of human health impacts and the number of cases per year is difficult because illnesses aren’t always reported or correctly attributed to HABs.^19^ In any case, these impacts would be considerably larger if not for robust state-level fishery monitoring programs, which are charged with ensuring that harvested fish and shellfish are safe for human consumption.^10^

Scientists don’t yet know how to predict specific blooms, but generally speaking, algal growth is related to nutrient levels in the water. Excess nitrogen content is often associated with more algae in marine waters, as is excess phosphorus in freshwater.^20^^,^^21^ Warmer waters are associated with blooms of toxin-producing cyanobacteria (also known as blue-green algae, although they are, in fact, bacteria); these blooms peak in lakes during summer months. In the long term, increasing average temperatures may allow certain species to expand their geographic ranges.^22^

However, it’s important for coastal managers and public health officials to know that the presence of an algal bloom doesn’t necessarily mean toxins are present. That’s because only a small percentage of algal species are capable of producing toxins, and production varies widely among those that do, says Greg Doucette, a research oceanographer with NOAA who has developed toxin sensors for the current and third-generation ESPs. “You can’t just *a priori* assume that those cells are going to be highly toxic, or minimally toxic, or moderately toxic,” he says. “The toxin levels can vary at least tenfold, and sometimes way more than that, depending on the toxin class and the species you’re talking about.”

That, Doucette says, is where the exciting part of this research is now starting to happen. “We’re starting to get these instruments out into the water, make these measurements, and see how [they relate] to the presence of toxins in the shellfish or the resource,” he says.

## Real-Time Detection

The U.S. government has recognized the value of developing such tools. To support research and authorize funding, Congress passed the Harmful Algal Bloom and Hypoxia Research and Control Act in 1998 and expanded it in 2004. A second reauthorization was signed by President Obama on 30 June 2014.^23^

**Figure:**
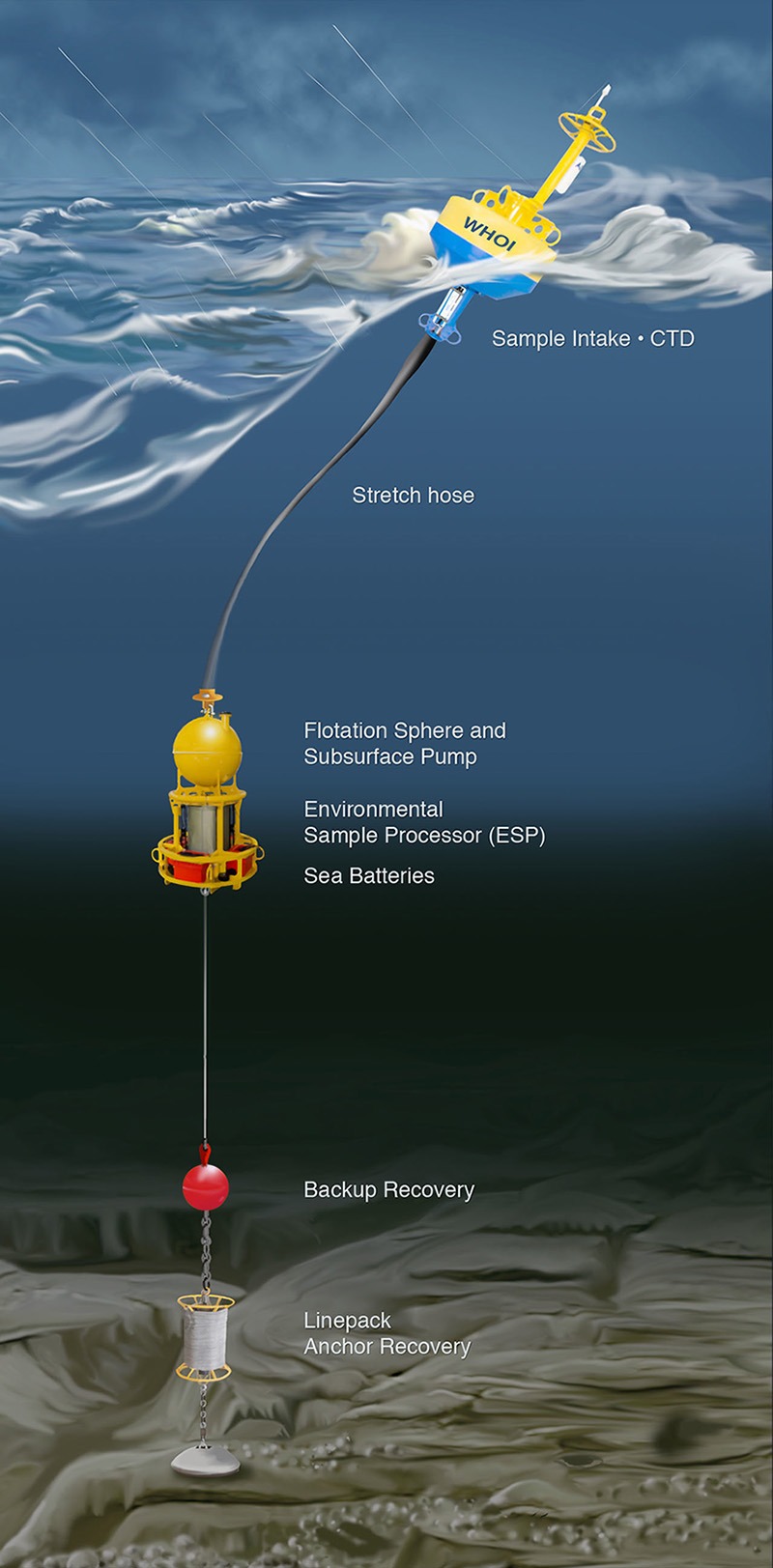
In a mooring configuration developed at Woods Hole Oceanographic Institution, the second-generation Environmental Sample Processor, or ESP, automatically collects and tests water samples for DNA and toxins that indicate the presence of HABs. © E. Paul Oberlander/WHOI

**Figure:**
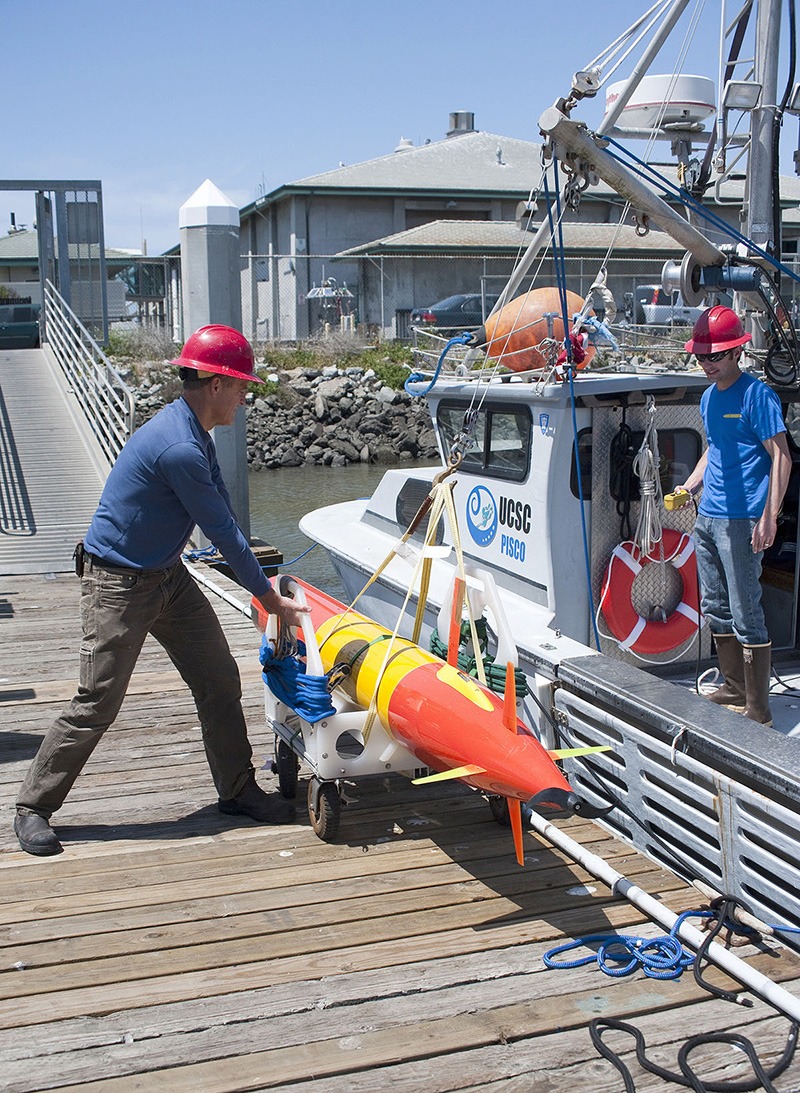
A much smaller third-generation ESP mounted within a long-range autonomous underwater vehicle will be able to physically follow and monitor developing blooms. The new ESP should be commercially available within three to four years. © Todd Walsh/MBARI

Yet despite this commitment and recent technological breakthroughs, funding for most research programs has declined due to the recession and the political atmosphere in Congress, says Don Anderson, a senior scientist and the HAB program lead at the Woods Hole Oceanographic Institution. “Times are definitely much more difficult now,” he says. “Everybody has had their budgets cut.”

The funds that do remain continue to support accelerated innovation in the field. Fred Tyson, who administers Oceans and Human Health (OHH) grants for the National Institute of Environmental Health Sciences (NIEHS), says this year’s Funding Opportunity Announcement received a high number of meritorious projects. Working creatively with the funds available to the institute and the National Science Foundation (which cosponsors the grants program), administrators were able to fund thirteen grantees instead of the anticipated four to six, most of them in the field of HAB science. Five of these were related directly to HAB detection.^24^

“Actual detection of HABs and forecasting of blooms is very important to NIEHS, because that allows us to employ prevention strategies,” Tyson says. “If people have a better way of detecting, then they have a better way of forecasting and providing the public with information they need to protect themselves.”

The five HAB detection projects funded this year employ a range of tactics and tools reflecting the various threats posed by HABs across the country. In the Gulf of Maine, researchers at the Woods Hole Center for Oceans and Human Health are testing another promising new instrument known as an Imaging FlowCytobot,^25^ an automated underwater microscope that can be used to rapidly detect *Alexandrium* cells by hitting them with a laser and then analyzing how they fluoresce or scatter light. The instrument—which, like the ESP, is commercially available and manufactured by McLane Research Laboratories—also takes high-resolution photos of cells and particles, allowing identification by species. The instrument is thus able to autonomously identify and enumerate HAB cells *in situ*.

**Figure:**
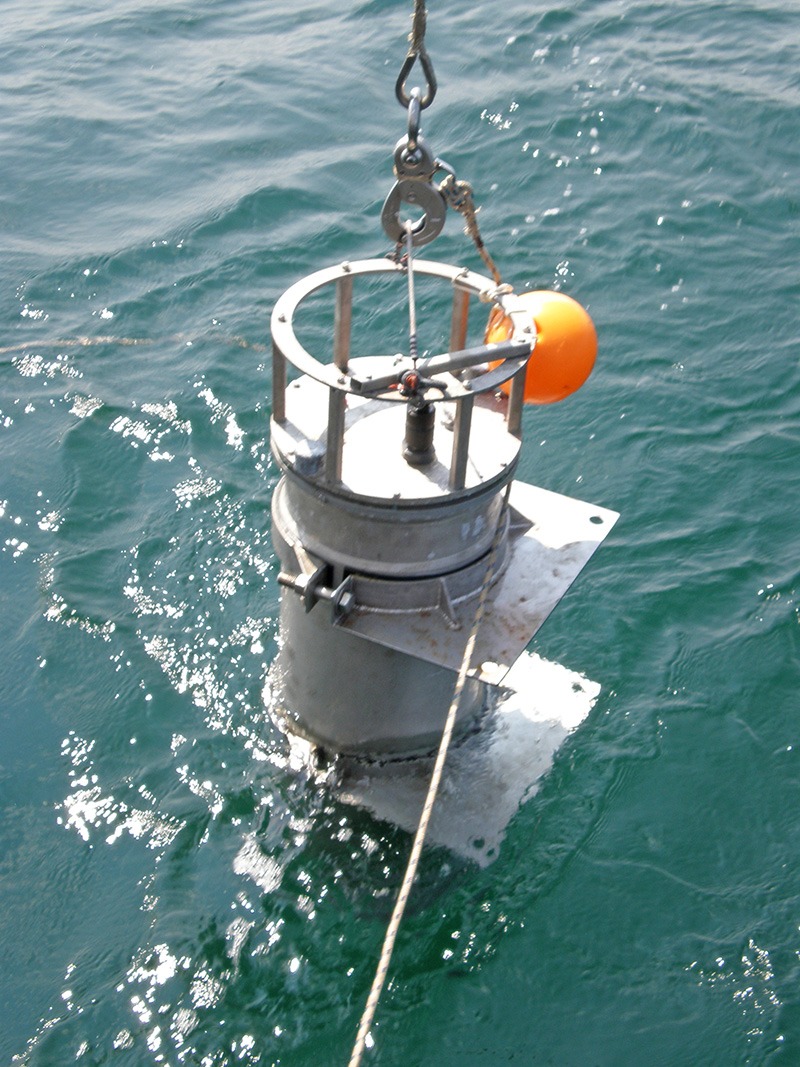
The Imaging FlowCytobot is an automated underwater microscope that produces high-resolution micrographs of particles suspended in the water. © McLane Research Laboratories

**Figure:**
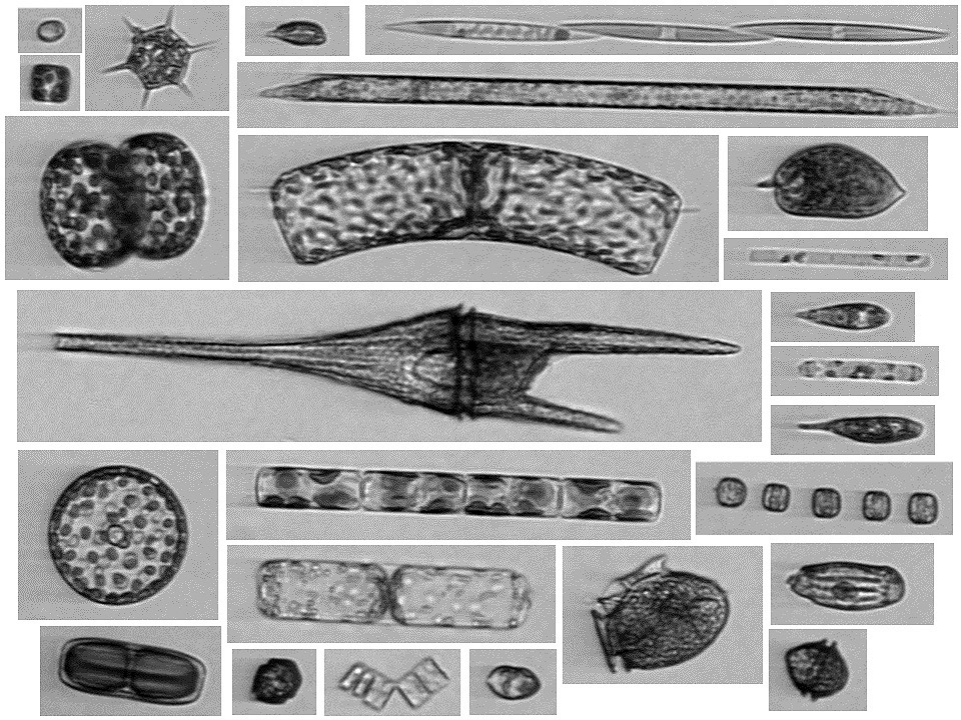
The images produced by the Imaging FlowCytobot are classified automatically with accuracy rivaling that of the trained human eye. © Heidi Sosik/WHOI

Although the Imaging FlowCytobot doesn’t detect toxicity, its benefits include rapid data processing, high-frequency sample collection and analysis (three samples per hour), multi-species identification capabilities, and the ability to be deployed at sea for up to six months at a time. It can also resolve different life stages of algal cells, which helps determine when blooms are going through major transitions—for instance, the formation of gametes, which fuse to create zygotes and then dormant cysts that fall to bottom sediments as seeds for future blooms.

The best use for the Imaging FlowCytobot may be in tandem with the ESP, according to Anderson. “It’s collecting very different data, but very complimentary data, too,” he says. In fact, the device has already proven itself as a management tool and in certain circumstances may be the more appropriate device, says NOAA program coordinator Quay Dortch. It has already been used for many years to provide HAB early warnings along the Texas coast.^26^

Three of this year’s OHH-funded projects target freshwater HABs in the Great Lakes. Hyeok Choi, an assistant professor of environmental engineering at the University of Texas at Arlington, received a grant for an *in situ* HAB toxin detection system targeted, at least initially, at Lake Erie. In recent years Lake Erie has seen increasingly troublesome blooms of cyanobacteria, which pose a significant threat to local ecosystems, fisheries, property values, recreation, tourism, and the drinking water of millions of Americans.^27^

Choi’s objectives include developing a tiny sensor that can detect targeted toxins at very low levels, constructing a localized wireless sensor network to allow the sensors to communicate with the lab, integrating the nanotechnology-enabled sensor and related parts into a coin-sized package, and installing the system at Lake Erie for a two- to three-month test. The first three steps will be complete by early 2015, and Choi hopes to have his sensors in the water for next summer’s bloom season. Compared with the top-of-the-line ESP, “our approach is a little bit different,” Choi says. “We don’t want to make such an expensive device. ... We can build the chip-like sensor very cheaply.”

Timothy Moore of the University of New Hampshire leads an OHH-funded team taking a very different approach. Their goal is to develop an algorithm combining water-quality data and remote-sensing imagery to predict toxic *Microcystis* blooms. For the past two summers, the team has deployed a buoy in Lake Erie to continuously measure water-quality parameters including temperature, phosphate concentration, oxygen content, algal fluorescence, and turbidity. The goal is to combine these limnological data with remote-sensing imagery of blooms in order to quantify their relationship and ultimately improve the detection and prediction capability of satellites. “We need to develop, test, and refine the algorithm on measurements we collect in the water before applying it to satellite data,” Moore says.

The third project aimed at freshwater HAB detection is led by Todd Miller, an assistant professor of environmental and occupational health at the University of Wisconsin–Milwaukee. His team seeks to use existing underwater sensors and an automated sampling device to study relationships between limnological variables in Lake Winnebago, near Lake Michigan, and toxins produced by cyanobacteria in the lake. They plan to use this information to develop a model that describes which toxins are likely to be present at what levels based on water-quality data. “That model may then give us an idea of when drinking-water plant managers need to worry about toxins breaking through their treatment systems,” Miller explains.

Finally, Judy Westrick, director of the Lumigen Instrument Center at Detroit’s Wayne State University, leads a team developing a HAB assay using a widely used method known as qPCR (quantitative polymerase chain reaction). qPCR works by identifying DNA segments; thus, in this context it is not toxins themselves that are being detected but rather the genes that would enable a microorganism to produce toxins. qPCR instruments are already employed by many public agencies for beach monitoring, Westrick says, and they could be adapted to run the new HAB assay. “From a human-health perspective, you could develop early warning,” she says. “Our goal was to be able to have this technique available to managers.”

## Better Forecasting

NOAA is another major source of funding for HAB research. Historically, its Oceans and Human Health Initiative has provided grants to scientists nationwide for work related to a breadth of water-quality issues, including HABs. However, in recent years the budget for this initiative has been completely cut.^28^

NOAA also manages a trio of competitive grant programs geared specifically toward HABs. The first grant program, known as ECOHAB (Ecology and Oceanography of Harmful Algal Blooms), was launched in 1998 to study the underlying causes and ecology of HABs. In one example of an ECOHAB project, a collaborative team led by the University of California, Santa Cruz, is studying how oceanographic processes and human activities influence HAB activity at two regional HAB hot spots (Monterey Bay in Central California and San Pedro Bay near Los Angeles) over a period of years. During April 2014 this team monitored HAB activity and ocean conditions off Southern California using a pair of ESPs, autonomous vehicles, ships, and numerical models. Three other ECOHAB projects were also funded in 2011, although no new awards have been allocated since then.^29^

The second grant program, MERHAB (Monitoring and Event Response for Harmful Algal Blooms), was launched in 1989 with the aim of moving science-based projects developed in ECOHAB or elsewhere into real-world, management-based settings, although it too has issued no new grants since 2011.^30^ Woods Hole’s Anderson is the lead investigator of one currently funded MERHAB project, which aims to incorporate data obtained from ESP units into existing HAB monitoring and management systems in the Gulf of Maine.

Anderson and colleagues hope to demonstrate how a network of ESPs can be used to augment the testing currently used to detect HAB toxins and trigger harvesting closures. ESP data can provide a greatly expanded view of the distribution of toxic organisms in a region. For instance, HAB threats along a coast are currently assessed by measuring toxins in shellfish using either animal bioassays or chemical methods such as high-performance liquid chromatography, Anderson explains. These samples are typically taken from shellfish collected along the shore, so no information is obtained about the algae themselves that may be present in near- or offshore waters. That information may now be available from the ESPs moored at sea. When combined with computer models of ocean circulation, the ESP data can greatly improve short-term forecasts that can help managers maximize the amount of time that shellfish beds can safely remain open for harvesting, Anderson says.

Finally, PCM HAB (Prevention, Control, and Mitigation of Harmful Algal Blooms) is a relatively new NOAA program designed to bring promising new detection and control technologies to coastal managers.^31^ The program is also concerned with assessing the economic impact of HABs.

PCM HAB currently funds Woods Hole’s forecasting program for *Alexandrium* in the Gulf of Maine. These forecasts rely on meteorological and oceanographic conditions of past years to predict the severity of the upcoming season. Between 2008 and 2013 the team’s predictions were accurate four times and wrong twice. For the 2014 season, they’ve predicted a moderate outbreak, which thus far seems to be accurate. Dortch, who manages the ECOHAB and PCM HAB programs, says the Gulf of Maine forecasts will eventually be transitioned into NOAA’s Harmful Algal Bloom Operational Forecast System, which already provides forecasts for *Karenia brevis* blooms off the coasts of Florida and Texas.^32^

## Turning Point

Despite steady funding cuts over the last decade, Dortch says HAB monitoring and detection technologies have arrived at a turning point. “It’s astonishing how many things are bearing fruit right now, in the sense that they’ve been developed and we can really start using them,” she says. “I think that we are really on the verge of many new capabilities, and I think it has been because of these programs.”

Still, despite the promise that emergent technologies hold for scientists and coastal managers, everyday beachgoers and boaters with their eyes on the water still play an important role in HAB monitoring, says Vera Trainer, manager of the Marine Biotoxin Program at NOAA’s Northwest Fisheries Science Center. Citizen-based monitoring programs (increasingly aided by smart-phone apps) also help keep tabs on HABs, with ordinary citizens trained to collect samples and perform simple microscope observations and counts at selected sites along a coast.

“These are essential parts of HAB monitoring in some regions,” Trainer says. “An integrated approach that combines citizen monitoring, standard shellfish monitoring, and new technologies will be the most comprehensive and arguably the most effective.”

After understanding and predicting HABs, there may yet be one final step: reducing human causes of blooms and fighting them when they’ve already begun. Korea and China are already experimenting with spraying clay on the water’s surface to aggregate and sink algal cells.^33^ Both countries have high-value fish-farming industries they are trying to protect from HABs. Don Anderson of Woods Hole Oceanographic Institution advocates for trying the approach in the United States. “It’s a very effective way of clearing cells from the water,” he says.

Some researchers and even the U.S. Environmental Protection Agency have explored the idea,^34^ and NOAA’s PCM HAB program is set up to fund some mitigation projects. Yet environmental regulations make it difficult to apply this sort of treatment in U.S. waters, Anderson says, and clay applications are impractical with large coastal blooms. For these big blooms, he says, the goal is still accurate forecasts to protect resources and ensure toxic fish and shellfish don’t get on the market.
